# Long-Read Assembly and a Within-Genus Structural Survey Reveal an Exceptionally Long Inverted Repeat in the *Rhododendron spinuliferum* Plastome

**DOI:** 10.3390/genes17091007

**Published:** 2026-08-26

**Authors:** Jingjing Xia, Ting Li, Yuxiang Pan, Dujuan Zhan, Xiaolei Sang, Alin Lin

**Affiliations:** 1 College of Agriculture and Biotechnology, Lishui University, Lishui 323000, China; xiajingjing@lsu.edu.cn (J.X.); ting_li01@126.com (T.L.); pyuxiang0719@126.com (Y.P.); 2 Industrial College of Traditional Chinese Medicine and Health, Lishui University, Lishui 323000, China; 3 School of Architecture, Huaqiao University, Xiamen 361021, China; 14309@hqu.edu.cn; 4 School of Civil Engineering and Architecture, Zhejiang Sci-Tech University, Hangzhou 310018, China; linalin@zstu.edu.cn

**Keywords:** *Rhododendron spinuliferum*, chloroplast genome, PacBio HiFi, inverted-repeat expansion, small single-copy region

## Abstract

**Background/Objectives**: Expanded inverted repeats (IRs) occur in *Rhododendron* plastomes, but their extent and evolutionary context remain incompletely resolved because repeat-rich genomes can be difficult to assemble from short reads. **Methods**: We assembled and curated the plastome of *Rhododendron spinuliferum* using publicly available PacBio HiFi reads and evaluated whether the same junction architecture was present using Illumina PE400 reads from a different individual. **Results**: Bandage-guided graph resolution and junction-spanning reads supported a 209,482 bp quadripartite plastome comprising a 108,321 bp large single-copy (LSC) region, a 2629 bp small single-copy (SSC) region, and two 49,266 bp IRs. All four inferred junctions were supported by HiFi reads and were also detected in the independent Illumina dataset, and short-read mapping covered every reference position without zero-depth sites. In a broad within-genus survey of 26 structurally resolved *Rhododendron* plastomes, *R. spinuliferum* had the longest validated conventional IR in the curated dataset. Twenty-four plastomes shared a 19-gene expanded-IR core and retained *ndhF* in the SSC, whereas *R. vialii* and *R. datiandingense* represented extreme SSC contraction and short-IR architecture, respectively. Ancestral-state reconstruction identified the expanded-IR core as the most likely crown state. After one IR copy was removed, repeat density was not correlated with IR length, and comparison with *R. delavayi* showed that the additional IR sequence in *R. spinuliferum* was distributed predominantly across intergenic regions. **Conclusions**: These results provide a validated plastome resource and clarify the structural evolution of expanded IRs in the sampled *Rhododendron* plastomes.

## 1. Introduction

Plastid genomes are widely used in plant systematics, population genetics, and comparative genomics because they retain a comparatively conserved gene set and order. Their canonical quadripartite organization consists of two inverted repeats (IRs) separated by large and small single-copy regions. Although this architecture is often conserved, it is not static. IR expansion and contraction can shift junctions, duplicate or exclude genes, and alter genome size and local sequence evolution. Repeat-mediated recombination can also produce alternative plastome arrangements. Therefore, an apparently circular assembly alone does not establish a structural evolutionary inference. Assembly graph inspection, long reads, and independent boundary validation are particularly important when plastome structure is interpreted as evidence of lineage history [1,2,3].

*Rhododendron* (Ericaceae) is a taxonomically complex genus in which plastomes show unusually broad structural variation. A long-read assembly of *Rhododendron delavayi* revealed a 46,968 bp IR, a 2610 bp small single-copy region, extensive repeats, and localized rearrangements. In parallel, a densely sampled plastome phylogeny resolved many relationships across the genus while also identifying conflicts with nuclear relationships. These studies show that plastome sequences are informative for lineage history. They also indicate that the structural configuration of a plastome may provide an additional, incompletely characterized set of evolutionary characters [3,4].

Recent comparative studies have extended the evidence for structural heterogeneity. Analyses of four *Rhododendron* plastomes, an 18-taxon comparison centred on *Rhododendron ambiguum*, and long-read analyses of two additional species all reported substantial IR-boundary variation or highly reduced small single-copy regions [5,6,7]. Together, these reports establish that IR expansion is not confined to a single exceptional accession. However, they were designed principally as individual genome reports or limited comparisons. Their results cannot, by themselves, establish the frequency of alternative IR architectures, their distribution on the sampled *Rhododendron* phylogeny, or whether the longest IRs represent the same boundary configuration. These questions require a structure-filtered dataset in which the two IR copies and their junctions are independently verified. The relationship among IR length, boundary-associated gene content, non-IR repeat landscape, and phylogenetic history has not been assessed together across such a *Rhododendron* dataset.

Here, we reconstructed the plastome of *Rhododendron spinuliferum* from publicly available PacBio HiFi reads, manually resolved the repeat graph, and independently evaluated the inferred junctions with Illumina reads from another individual. We then surveyed 26 structurally resolved *Rhododendron* plastomes to identify IR-boundary architectures and reconstruct the history of IR and small single-copy traits. We further tested whether the exceptional IR of *R. spinuliferum* reflects the extension of a shared expanded-IR configuration or the accumulation of lineage-specific sequence. This was evaluated by integrating boundary gene content, IR self-alignment, one-IR-removed repeat analyses, comparative collinearity, and phylogenetic trait mapping. By separating read-supported structural evidence from annotation-derived coordinates, this study provides a validated plastome resource and a comparative framework for interpreting IR expansion in *Rhododendron*. It also offers a cautious basis for distinguishing biologically meaningful structural variation from artefacts of repeat-rich plastome assembly.

## 2. Materials and Methods

### 2.1. Sampling and Sequencing

Fresh leaves of *R*. *spinuliferum* were collected from Zixi Mountain National Forest Park, Chuxiong, Yunnan Province, China (25.05° N, 101.43° E), on 15 March 2026. A voucher specimen (LSU_20260312) was deposited at the Herbarium of Lishui University, China. Total genomic DNA was extracted from leaf tissue using the Rapid Plant Genomic DNA Isolation Kit (Sangon Biotech, Shanghai, China). An Illumina paired-end library was prepared from this individual and generated 11,213,581 pairs of 151 bp reads, representing approximately 3.39 Gb of sequence data. Because the repeat-rich and structurally complex plastome could not be resolved from the in-house short reads alone, publicly available PacBio HiFi reads from a different *R. spinuliferum* individual were retrieved from the Genome Sequence Archive (GSA) at the National Genomics Data Center (NGDC) under accession CRR1094297 and used for the primary de novo assembly. Accordingly, the submitted plastome sequence PZ603433 represents the public HiFi individual rather than the Zixi Mountain voucher specimen; the voucher-linked Illumina dataset was used for independent structural comparison.

### 2.2. Assembly and Annotation of the Chloroplast Genome

PacBio HiFi reads were mapped against complete *Rhododendron* chloroplast genomes to recruit plastid reads and were assembled de novo with Flye v2.9.6 [8]. For read recruitment, complete plastomes from the genus were used as bait; HiFi alignments were generated with minimap2 v2.24-r1122 in map-hifi mode, and reads were retained with a mapping quality of at least 20, an aligned length of at least 1000 bp, and an aligned fraction of at least 0.50. A high-confidence plastid contig was then used for iterative recruitment with a mapping quality of at least 20 and an aligned length of at least 1000 bp; a 4000-read subset of the resulting reads was assembled in PacBio HiFi mode with a genome size of 220 k, assembly coverage of 120, and one iteration. The assembly graph was inspected in Bandage v0.8.1 [9], and the final circular chloroplast genome was manually resolved from the GFA graph and HiFi spanning-read evidence across the four junctions. HiFi reads were mapped to synthetic junction references with minimap2 v2.24 [10]; a read was counted as junction-supporting when it spanned at least 1 kb on both sides of the junction center. HiFi reads were additionally screened against paired local IRb-SSC-IRa references representing the two SSC orientations; reads spanning the complete SSC and at least 1 kb of both IR flanks were counted, and the Flye GFA was inspected for unexpected graph links. The in-house Illumina reads were mapped to 300 bp junction references, and single-read support required at least 50 bp on each side. Because the Illumina and PacBio datasets originated from different individuals, the Illumina reads were used to test whether the same species-level structural configuration and junction architecture were present in the Zixi Mountain individual; they were not used for sequence polishing or correction of the HiFi assembly. The chloroplast genome was annotated with GeSeq v2.03 [11]. tRNA genes were checked with tRNAscan-SE v2.0.9 [12] and ARAGORN v1.2.38 [13]. Gene boundaries, intron-containing genes, and IR/LSC/SSC boundaries were manually curated by comparison with closely related *Rhododendron* plastomes. The finalized chloroplast genome was submitted to GenBank under accession PZ603433. For whole-plastome coverage analysis, the same PE400 reads were mapped to the final manually curated 209,482 bp circular plastome assembly deposited as PZ603433 (local reference file: final_manual_circular_candidate.fa), not to an intermediate contig, draft path, or graph-derived sequence.

### 2.3. Chloroplast Genome Characteristics

We calculated total genome length, GC content, and the lengths of the LSC, SSC, and two IR regions from the finalized plastome with SeqKit v2.1.0 [14]. The circular chloroplast map was generated with OGDRAW v1.3.1 [15] from the curated annotation and displays the LSC, SSC, IRa, and IRb regions with standard gene-feature tracks.

### 2.4. Repeat and SSR Analyses

Forward, reverse, complement, and palindromic interspersed repeats were detected with Vmatch v2.3.1 [16], using a minimum repeat length of 30 bp, a maximum length of 100 bp, and up to three mismatches. This focal-genome repeat analysis was restricted to the complete plastome and was used only for the descriptive repeat/SSR profile, not for cross-taxon repeat-density comparisons. Simple sequence repeats (SSRs) were identified with MISA v2.1 [17] using minimum repeat thresholds of 10, 5, 4, 3, 3, and 3 for mono-, di-, tri-, tetra-, penta-, and hexanucleotide motifs, respectively. Adjacent SSRs separated by no more than 100 bp were classified as compound SSRs.

### 2.5. Analysis of Codon Preference

Codon-usage statistics and relative synonymous codon usage (RSCU) values were calculated with CodonW v1.4.4 using annotated protein-coding sequences. The bacterial, archaeal, and plant plastid genetic code (translation table 11) was applied, stop codons were excluded, and genes with incomplete or ambiguous coding regions were omitted. RSCU profiles were visualized in R v4.1.2 for comparative interpretation.

### 2.6. Analysis of IR Boundaries

The IR/LSC/SSC boundaries of *R. spinuliferum* and four representative, publicly available *Rhododendron* plastomes were compared with IRscope v3.1 [18]. The comparison taxa were selected based on whole-plastome similarity, complete and curated annotations, and representation of different IR-size classes; their accessions are listed in Table S1. Curated GenBank files were uploaded to IRscope to visualize the JLB, JSB, JSA, and JLA junctions and to record boundary positions, gene overlaps, and partial-gene extensions in the LSC, SSC, IRa, and IRb regions.

### 2.7. Comparative Plastome Alignment, Nucleotide Diversity, and Ka/Ks Analyses

The four representative *Rhododendron* plastomes were aligned to the *R. spinuliferum* plastome in mVISTA using Shuffle-LAGAN mode [19]. Whole-plastome multiple alignments were then used to calculate nucleotide diversity (Pi) in DnaSP v6 [20] with a 600 bp sliding window and a 200 bp step; gaps and missing sites were treated as missing data. Windows at or above the 95th percentile of Pi were designated as high-variability windows for downstream reporting. For regional summaries, a window was assigned to the LSC, IRb, SSC, or IRa only when all reference positions in the 600 bp interval fell within that partition; windows spanning a regional boundary were classified separately as junction windows and were not assigned by midpoint.

For selection-pressure analysis, *R. spinuliferum* was used as the reference, and the same four representative *Rhododendron* plastomes were used as comparison taxa. Orthologous single-copy protein-coding sequences shared by all taxa were extracted with PhyloSuite v1.2.1 [21]. Putative orthologs were identified by reciprocal best BLASTP hits, translated proteins were aligned with MAFFT v7 [22], and codon-aware nucleotide alignments were generated from the protein alignments. Coding sequences with frameshifts, ambiguous bases, internal stop codons, or unreliable alignments were excluded, and low-confidence columns were removed with Gblocks v0.91b [23]. Pairwise Ka and Ks values were estimated using the MYN method in KaKs_Calculator 3.0 [24] under translation table 11. Ka/Ks values were reported as NA when Ks equalled zero or no estimate was returned, and genes with Ks greater than 2 were excluded from biological interpretation. Values of Ka/Ks greater than 1 were not interpreted as evidence of positive selection without additional site-model support.

### 2.8. Phylogenetic Relationship Analysis

Thirty-two taxa, including 15 *Rhododendron* taxa, representatives of the major Ericaceae lineages, and the two non-Ericaceae outgroups *Actinidia chinensis* and *Camellia sinensis*, were used for phylogenetic inference; taxa and accessions are provided in Table S1. Thirty-five orthologous single-copy protein-coding genes shared among all taxa were individually aligned with MAFFT v7 [22], concatenated into a 29,691 bp matrix, and partitioned by locus group. A maximum-likelihood tree was inferred under partition-specific best-fitting models selected by ModelFinder [25] in IQ-TREE v2.0.7 [26], with 1000 ultrafast bootstrap replicates [27]. The final topology and node supports were visualized with ggtree [28].

### 2.9. Within-Genus IR/SSC Structural Survey and IR Gene-Content Classification

On 18 July 2026, 32 publicly available species-level *Rhododendron* plastome records and the curated *R. spinuliferum* plastome (PZ603433) were screened. One representative record was retained per public species. IR coordinates were inferred from GenBank repeat features where available and independently validated by whole-plastome BLAST+ self-alignments [29]. Only plastomes with a resolved, conventional quadripartite structure and a validated inverted-repeat pair were retained for the primary analysis. For each retained plastome, the 19-gene expanded-IR core and the boundary marker *ndhF* were localized by nucleotide similarity searches against the complete sequence; a locus was considered IR-localized only when two complete homologs fell within the validated IR pair. The excluded records, accession numbers, and record-specific exclusion reasons are listed in Table S2.

### 2.10. IR/SSC Trait Mapping and Ancestral-State Reconstruction

For phylogenetic mapping of IR/SSC evolution in the sampled *Rhododendron* plastomes, the 26 structurally resolved *Rhododendron* plastomes and *Vaccinium bracteatum* were analyzed using 50 shared single-copy protein-coding genes. Genes were aligned with MAFFT v7 [22], concatenated into a 38,668 bp matrix, and analyzed under partition-specific models selected by ModelFinder [25] in IQ-TREE 2 [26] with 1000 ultrafast bootstrap replicates [27]. The tree was rooted with *V. bracteatum* before pruning the outgroup for visualization. IR and SSC lengths were reconstructed separately by maximum likelihood under a Brownian-motion model in ape::ace [30]. The observed boundary architecture was additionally reconstructed as a three-state discrete trait under an equal-rates model. The equal-rates model was used as a conservative exploratory model because two of the three observed structural states were represented by few tips, making more parameter-rich transition models poorly identifiable. Confidence intervals for continuous ancestral estimates were obtained from the ace profile likelihood; normalized likelihoods for discrete states are reported as relative likelihoods. *V. bracteatum* was used only to determine the root position; the ape::ace reconstructions were fitted to the 26 *Rhododendron* tips after the outgroup had been pruned. The reported intervals are conditional profile-likelihood intervals under the fitted Brownian-motion model and fixed topology and branch lengths, and they do not incorporate uncertainty in topology, branch lengths, model choice, or outgroup sampling. As a root-sensitivity check, the continuous-trait reconstruction was repeated after pruning *V. bracteatum* before rooting and rerooting the *Rhododendron*-only tree at an alternative ingroup midpoint split.

### 2.11. One-IR-Removed Interspersed Repeat Landscape

One verified IR copy was removed from each structurally resolved plastome before repeat calling to prevent the canonical IR pair from dominating the repeat landscape. The artificial termini created by the deleted IR were treated as sequence ends and were not interpreted as internal repeat junctions. Nonredundant supermaximal repeats were called with Vmatch v2.3.1 [16] using -l 30, -h 3, and -supermax, classified as forward, palindromic, reverse, or complement pairs, and normalized as repeat pairs per 100 kb of the one-IR-removed sequence. This internally consistent 26-taxon repeat set had no upper repeat-length limit and was used for all cross-taxon repeat-density comparisons, including the value reported for the focal plastome. The association between IR length and normalized repeat-pair density was evaluated with Spearman’s rank correlation.

### 2.12. Focused Comparison of the Long IRs in R. spinuliferum and R. delavayi

The validated IR sequences of *R. spinuliferum* and the long-read *R. delavayi* plastome (MN711645) were aligned with progressiveMauve v2.4.0 [31]. Locally collinear backbone coverage and sequence not assigned to the shared backbone were calculated separately for each IR. Non-collinear blocks were intersected with the curated *R. spinuliferum* annotation to assess whether the IR-length difference was associated with complete gene capture, tRNA-associated regions, or predominantly intergenic sequence. Mauve blocks were interpreted as alignment categories and were not called insertions without breakpoint-level evidence.

## 3. Results

### 3.1. Assembly, Annotation, and Overall Plastome Features

**Figure 1:**
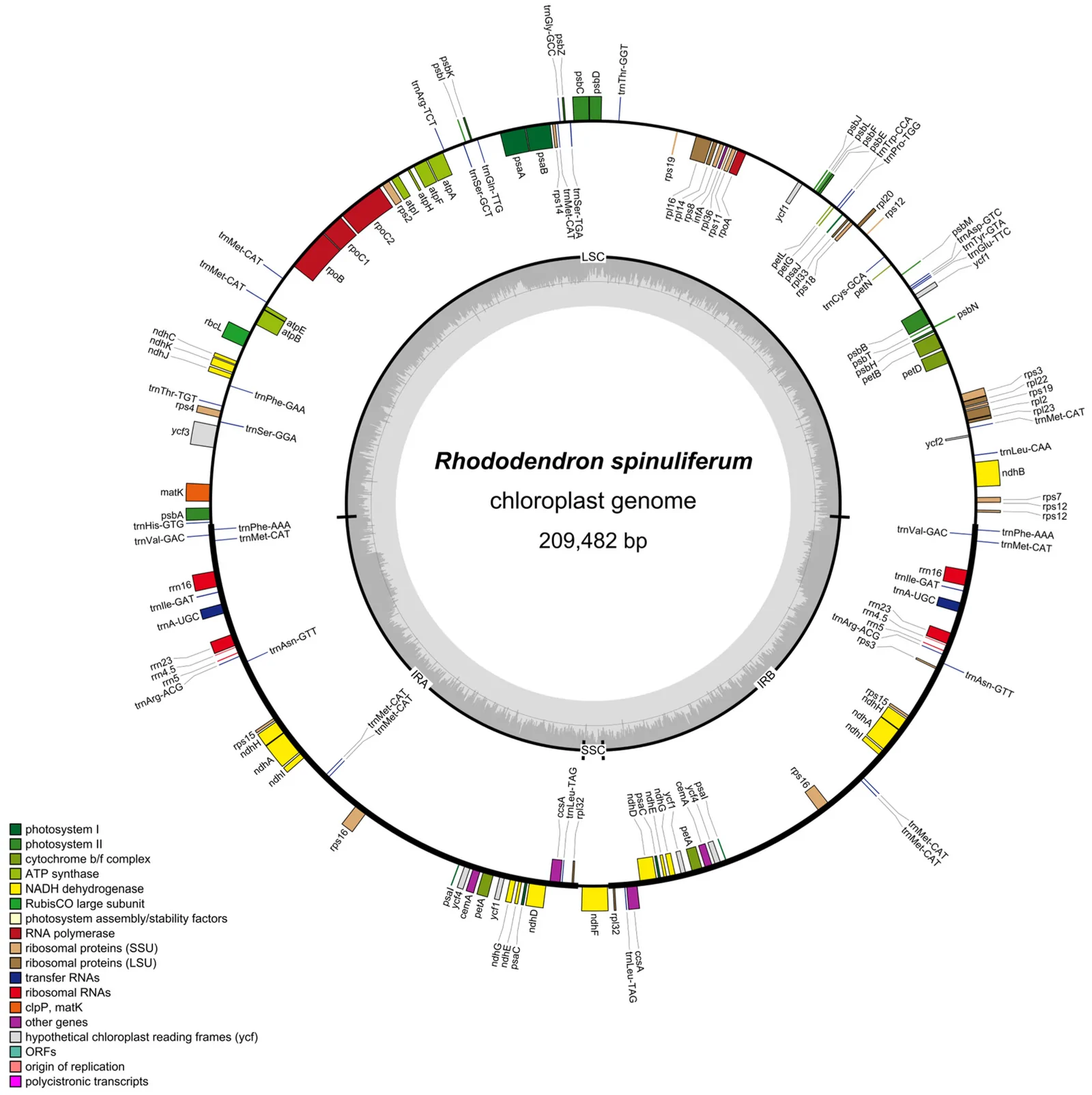
Circular chloroplast genome map of *Rhododendron spinuliferum*. The LSC, SSC, IRa, and IRb partitions and the inner GC-content track are shown.

Long-read graph curation and junction-spanning HiFi reads supported a circular, quadripartite *R. spinuliferum* plastome of 209,482 bp (PZ603433). The genome comprises a 108,321 bp LSC, a 2629 bp SSC, and two 49,266 bp IRs, with an overall GC content of 35.92%. The curated annotation contains 146 gene copies, including 97 protein-coding genes, 41 tRNA genes, and 8 rRNA genes (Figure 1; Table 1). All four manually resolved junctions were supported by 1036–2675 HiFi reads spanning at least 1 kb on each side; independent PE400 reads supplied 148–193 single-read supports spanning at least 50 bp on each side (Figure S1). An additional SSC-orientation screen detected 651 HiFi reads spanning the complete SSC and at least 1 kb of both IR flanks (765 reads at a 500 bp flank threshold). These reads supported local IR-SSC-IR continuity but could not yield a reliable SSC-isomer ratio, and the Flye graph showed no unexpected local junctions. Independent PE400 mapping covered all 209,482 reference positions without zero-depth bases (median depth 578×; mean depth 572.52×; Figure S2). This coverage calculation used the final manually curated circular assembly deposited as PZ603433 as the mapping reference.

### 3.2. Repeat, SSR, and Codon-Usage Patterns

**Figure 2:**
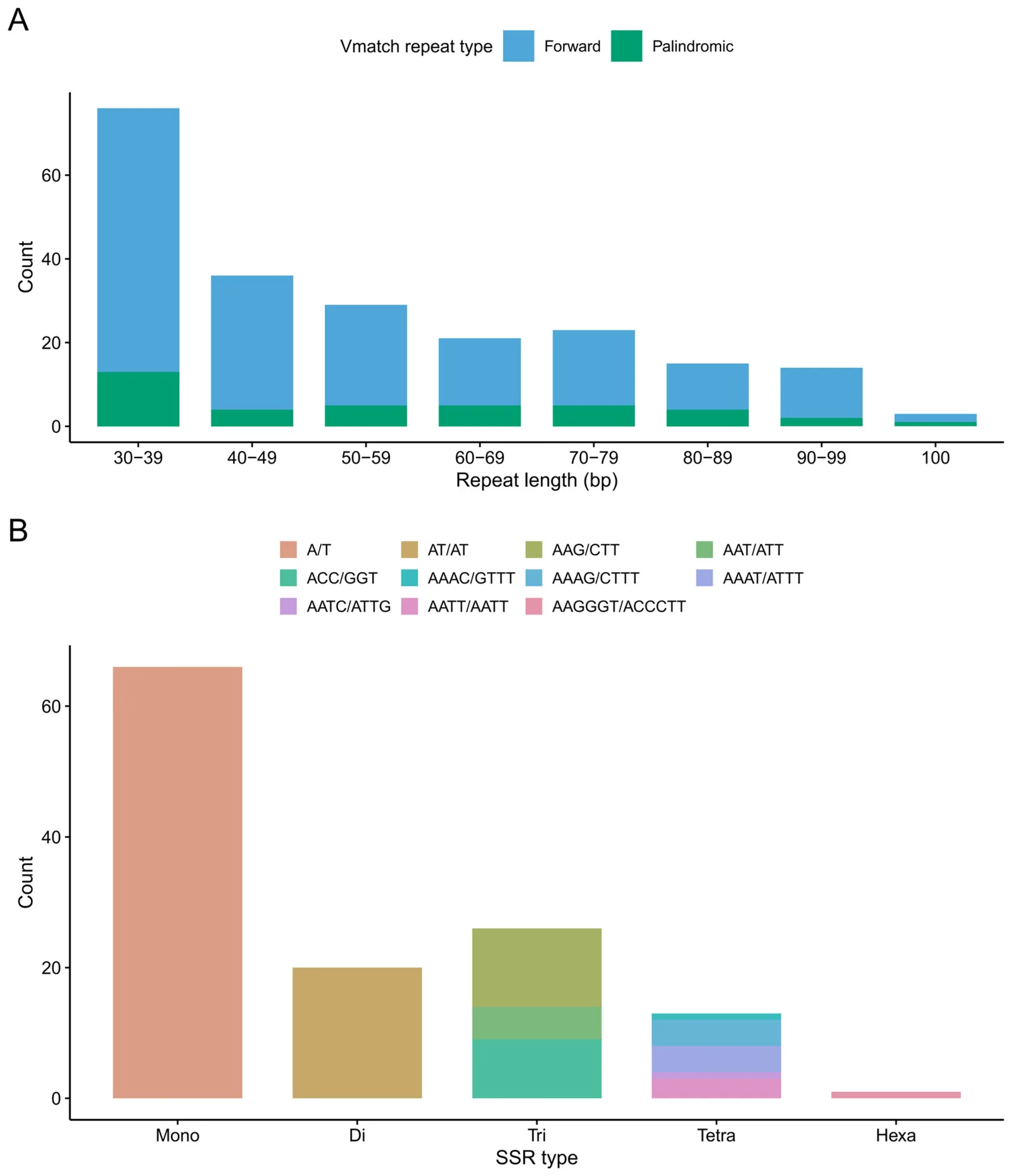
Interspersed-repeat and simple sequence repeat (SSR) profiles of the *Rhododendron spinuliferum* plastome. (**A**) Length distribution of forward and palindromic interspersed repeats detected with Vmatch. The interspersed-repeat panel shows the 30–100 bp complete-plastome repeat set. (**B**) Distribution of MISA SSRs by repeat class and motif category.

Vmatch detected 217 interspersed repeat pairs in the *R. spinuliferum* plastome, comprising 178 forward and 39 palindromic pairs. No reverse or complement pairs met the selected thresholds. This 217-pair catalogue reflects the 30–100 bp complete-plastome settings and is not directly comparable with the one-IR-removed supermaximal-repeat counts reported below. MISA identified 126 SSRs, including 66 mononucleotide, 20 dinucleotide, 26 trinucleotide, 13 tetranucleotide, and 1 hexanucleotide locus; 24 SSRs occurred in compound configurations. The SSR set was dominated by A/T-rich mononucleotide motifs (Figure 2). The RSCU profile indicated preferential use of A/T-ending synonymous codons. The most overrepresented codons were TTA for leucine (RSCU = 2.10), TCT for serine (1.96), and GCT for alanine (1.83); 29 codons had RSCU values above 1.0 (Figure S3).

### 3.3. IR/SSC Boundary Comparison

**Figure 3:**
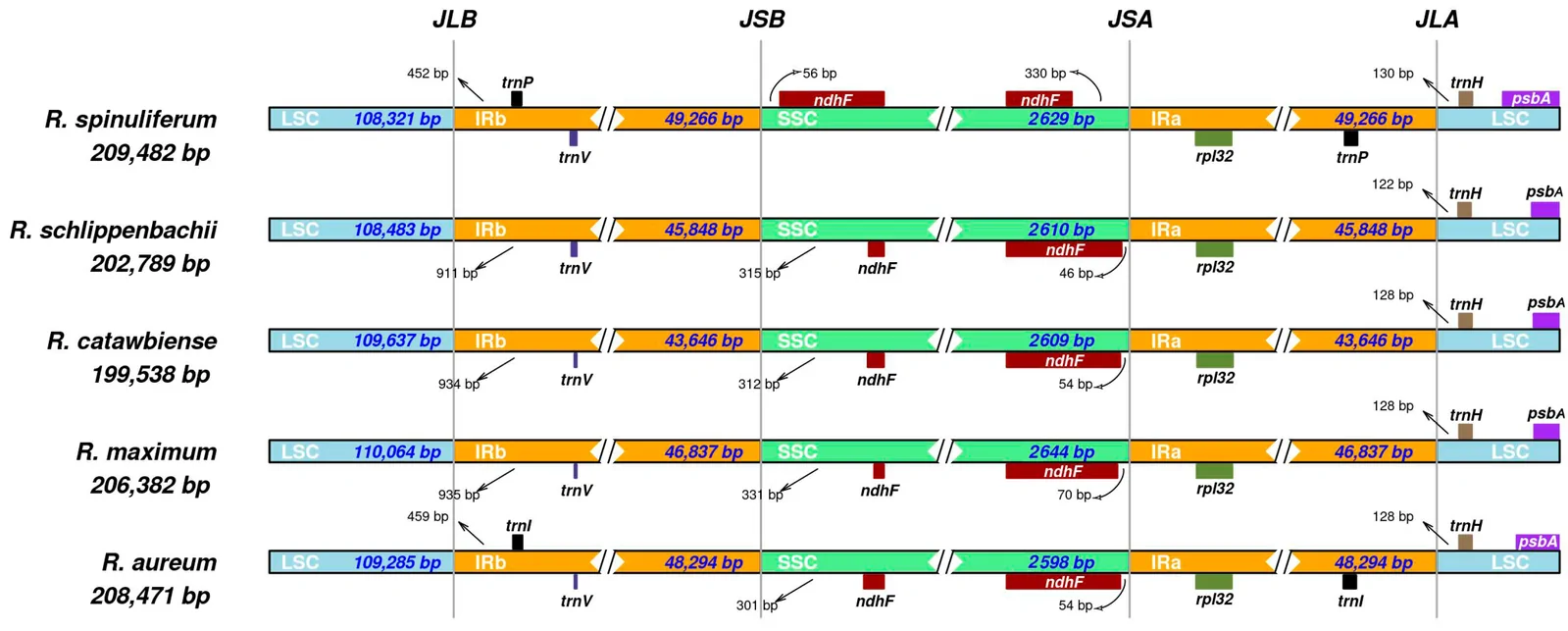
Comparison of LSC/IR/SSC junctions among *Rhododendron spinuliferum* and four representative *Rhododendron* plastomes. Arrows indicate the position and orientation of genes adjacent to the JLB, JSB, JSA, and JLA boundaries. Numbers next to boundary features indicate the distance or gene extension in base pairs.

The five-taxon IRscope comparison showed a shared expanded-IR and short-SSC architecture, but *R. spinuliferum* had the longest IRs (49,266 bp) among the selected taxa. Its SSC was 2629 bp, and *ndhF* remained closely associated with the IR/SSC junctions. The selected comparator plastomes had IR lengths of 43,646–48,294 bp and similarly short SSC regions (2598–2644 bp), indicating that the exceptionally long *R. spinuliferum* plastome principally reflects further IR expansion rather than an unusually long SSC (Figure 3).

### 3.4. Comparative Plastome Divergence, Nucleotide Diversity, and Ka/Ks Analyses

**Figure 4:**
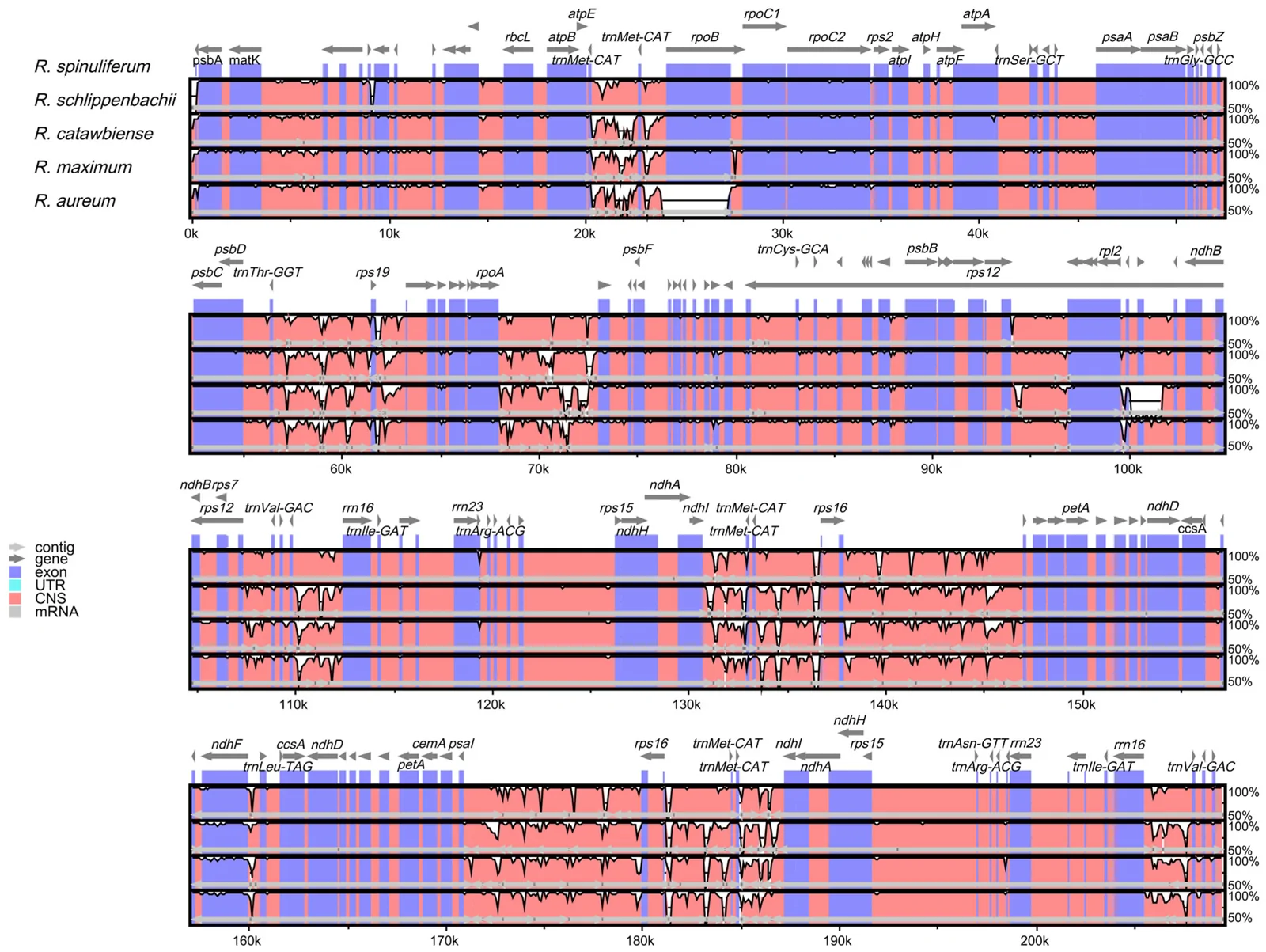
mVISTA comparison of four representative *Rhododendron* plastomes using *R. spinuliferum* as the reference. Percent identity is shown for *R. schlippenbachii*, *R. catawbiense*, *R. maximum*, and *R. aureum*. Gry arrows indicate annotated gene orientation; blue blocks indicate exons, and red blocks indicate conserved non-coding sequence.

mVISTA comparison of the five plastomes showed that most coding regions were conserved, whereas several intergenic regions and boundary-associated loci exhibited greater divergence (Figure 4).

**Figure 5:**
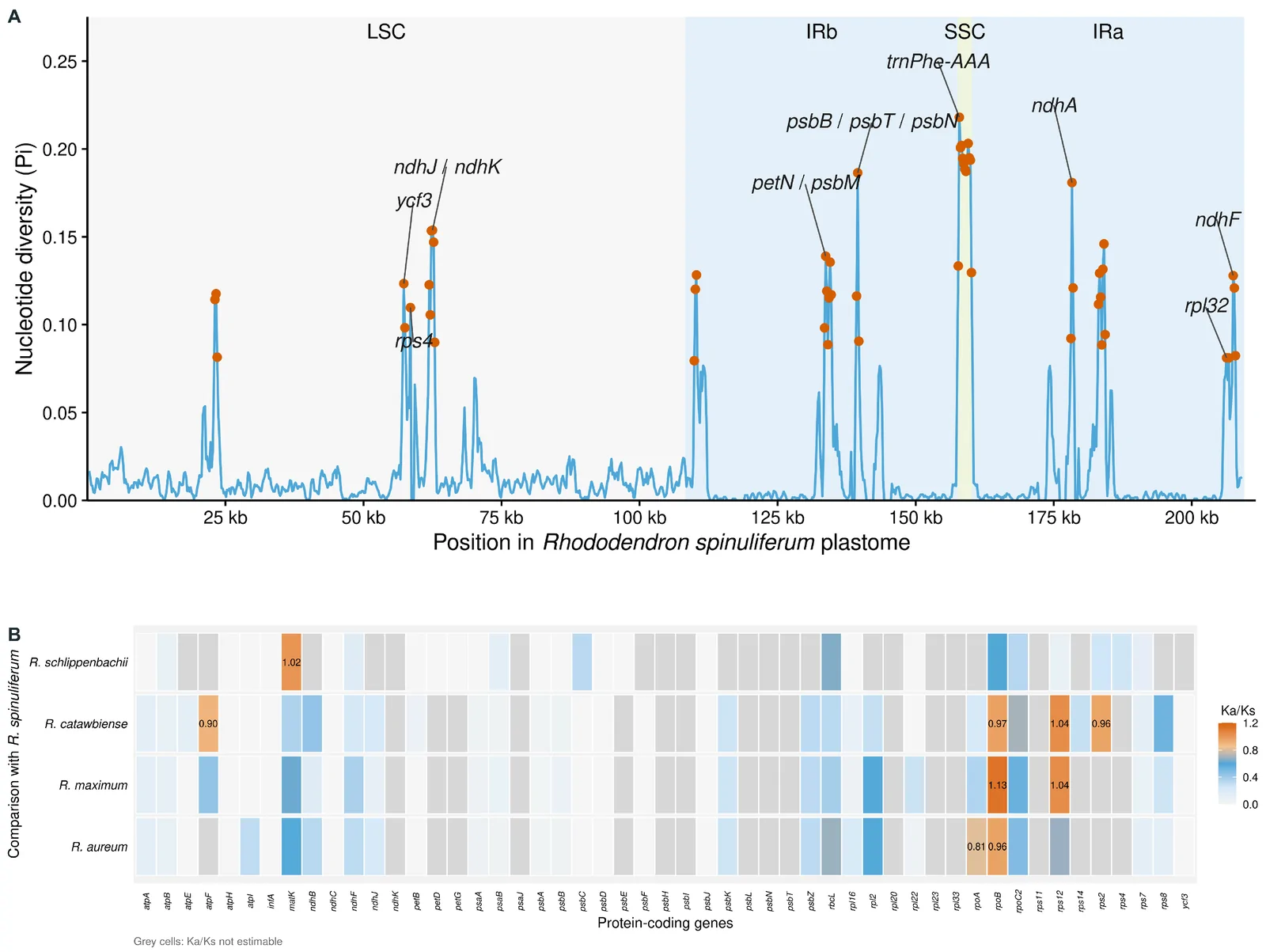
Sequence divergence and coding-sequence evolutionary rates among *Rhododendron spinuliferum* and four representative *Rhododendron* plastomes. (**A**) Sliding-window nucleotide diversity (Pi) was calculated across the five-plastome alignment using a 600 bp window and 200 bp step. Shaded bands identify the LSC, IRb, SSC, and IRa regions; orange points indicate windows at or above the 95th percentile of Pi. Region labels for window summaries used strict containment, and boundary-spanning windows were classified as junction windows. (**B**) Pairwise Ka/Ks heatmap for *R. spinuliferum* and the four representative plastomes. Values were estimated for 51 shared, single-copy, quality-controlled protein-coding genes with the MYN method in KaKs_Calculator 3.0. Gry cells indicate that Ka/Ks was not estimable and were not converted to zero. Zero-valued cells indicate true Ka = 0 estimates. Values at or above 0.80 are shown in the corresponding cells; values above 1.0 were not interpreted as evidence of positive selection without additional codon-site or branch-site tests.

Across the complete plastome alignment, nucleotide diversity (Pi) was 0.01537, and the 95th percentile of sliding-window Pi was 0.07737. The 1045 sliding windows comprised 539 LSC, 243 IRb, 11 SSC, 243 IRa, and nine junction windows. The junction windows included three LSC/IRb, three IRb/SSC, and three SSC/IRa windows; thus, the SSC summary was based on 11 wholly SSC-contained windows, with boundary-overlapping windows treated separately. High-Pi clusters were located near *ycf3*, *ndhJ*/*ndhK*, *psbB*/*psbT*/*psbN*, *ndhA*, and *ndhF* (Figure 5A). The highest Pi values occurred within the unusually short SSC region (2629 bp). Given its limited length and boundary displacement associated with extensive IR expansion, these peaks should be interpreted cautiously rather than treated automatically as conventional hypervariable-locus signals.

Of 58 shared single-copy protein-coding genes, 54 were identified as reciprocal-best-BLASTP orthologs, and 51 passed codon-alignment quality control. These genes yielded 204 pairwise comparisons between *R. spinuliferum* and the four comparator plastomes, of which 131 produced estimable Ka/Ks values; no comparison had Ks > 2. Median Ka/Ks values were 0.000, 0.129, 0.103, and 0.128 for *R. schlippenbachii*, *R. catawbiense*, *R. maximum*, and *R. aureum*, respectively. In the *R. schlippenbachii* comparison, the 0.000 median arose from true zero nonsynonymous-rate estimates: 16 of the 51 quality-controlled genes returned Ka = 0 and Ka/Ks = 0, 12 returned non-zero estimable values, and 23 were recorded as NA; because 16 of the 28 estimable values were zero, the median was zero. Four isolated estimates slightly exceeded 1.0: *matK* in the comparison with *R. schlippenbachii*, *rps12* with *R. catawbiense* and *R. maximum*, and *rpoB* with *R. maximum*. These isolated values were not interpreted as evidence of positive selection because no codon site-model test was performed (Figure 5B).

### 3.5. Phylogenetic Placement

**Figure 6:**
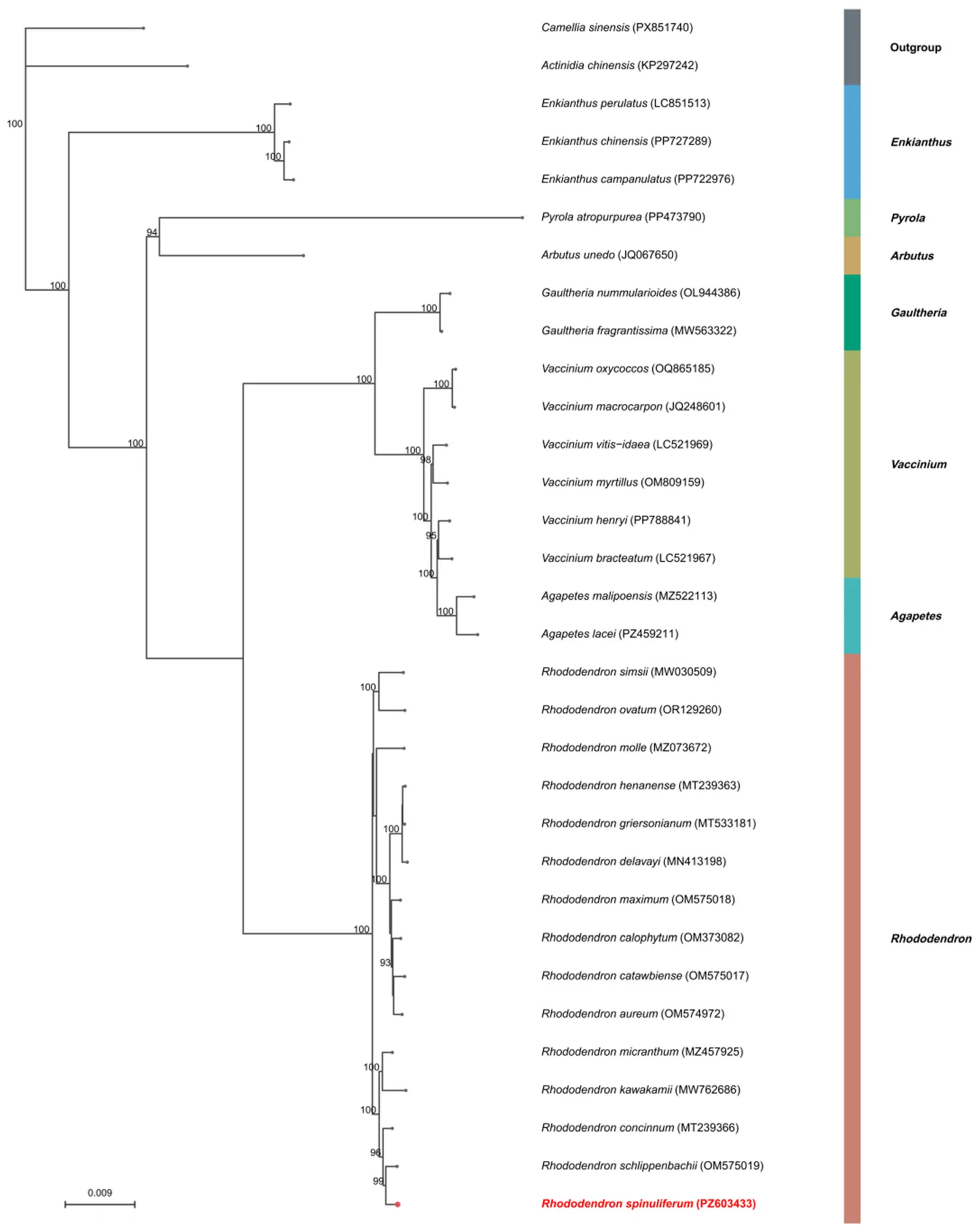
Maximum-likelihood phylogeny inferred from a concatenated alignment of 35 shared single-copy protein-coding genes. Node labels show ultrafast bootstrap support. *Rhododendron spinuliferum* is highlighted in red, and colored bars indicate generic membership. *Actinidia chinensis* and *Camellia sinensis* were used as outgroups.

The 35-gene, 29,691 bp coding-sequence matrix recovered a well-supported *Rhododendron* clade. *R. spinuliferum* was resolved as sister to *R. schlippenbachii* with 99% ultrafast bootstrap support, and this pair was placed with *R. concinnum* in a strongly supported lineage (Figure 6).

### 3.6. Within-Genus IR/SSC Structural Diversity

**Figure 7:**
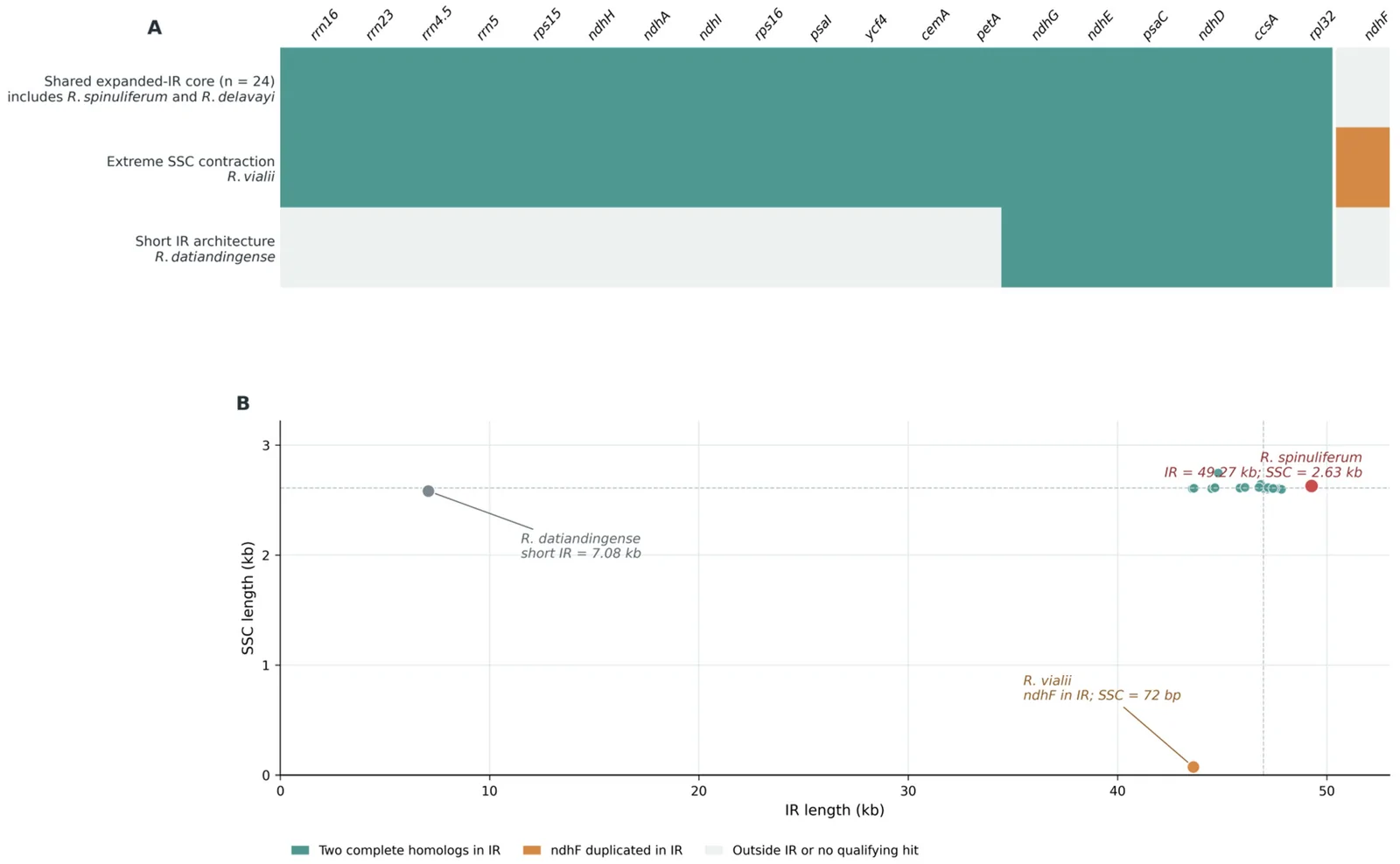
Within-genus IR boundary gene-content architectures in 26 structurally resolved *Rhododendron* plastomes. (**A**) Sequence-validated localization of 19 expanded-IR core genes and the boundary marker *ndhF*. Twenty-four plastomes, including *R. spinuliferum* and *R. delavayi*, share the expanded-IR core and retain *ndhF* in the SSC. *R. vialii* has *ndhF* duplicated in the IR, whereas *R. datiandingense* has the short-IR architecture. (**B**) Distribution of IR and SSC lengths for the three structural types. *R. spinuliferum* is highlighted in red.

Among 33 screened records, 26 structurally resolved plastomes were retained for the primary within-genus survey and 7 records were excluded because their IR/SSC architecture could not be resolved confidently. *R. spinuliferum* possessed the longest validated conventional IR in this curated, structurally resolved dataset (49,266 bp); the next longest was *R. brachycarpum* (47,826 bp), followed by the long-read *R. delavayi* plastome (46,968 bp). An amount of 24 of the 26 plastomes shared the same sequence-validated 19-gene expanded-IR core and retained *ndhF* in the SSC, including *R. spinuliferum* and *R. delavayi*. In contrast, *R. vialii* had *ndhF* duplicated in the IR and a 72 bp SSC, whereas *R. datiandingense* represented the short-IR architecture (Figure 7). Because each less frequent architecture was represented by a single retained plastome, interpretations of these rare structural types were treated cautiously. Record-specific exclusion reasons are provided in Table S2.

### 3.7. Phylogenetic Mapping and Ancestral-State Reconstruction of IR/SSC Architecture

**Figure 8:**
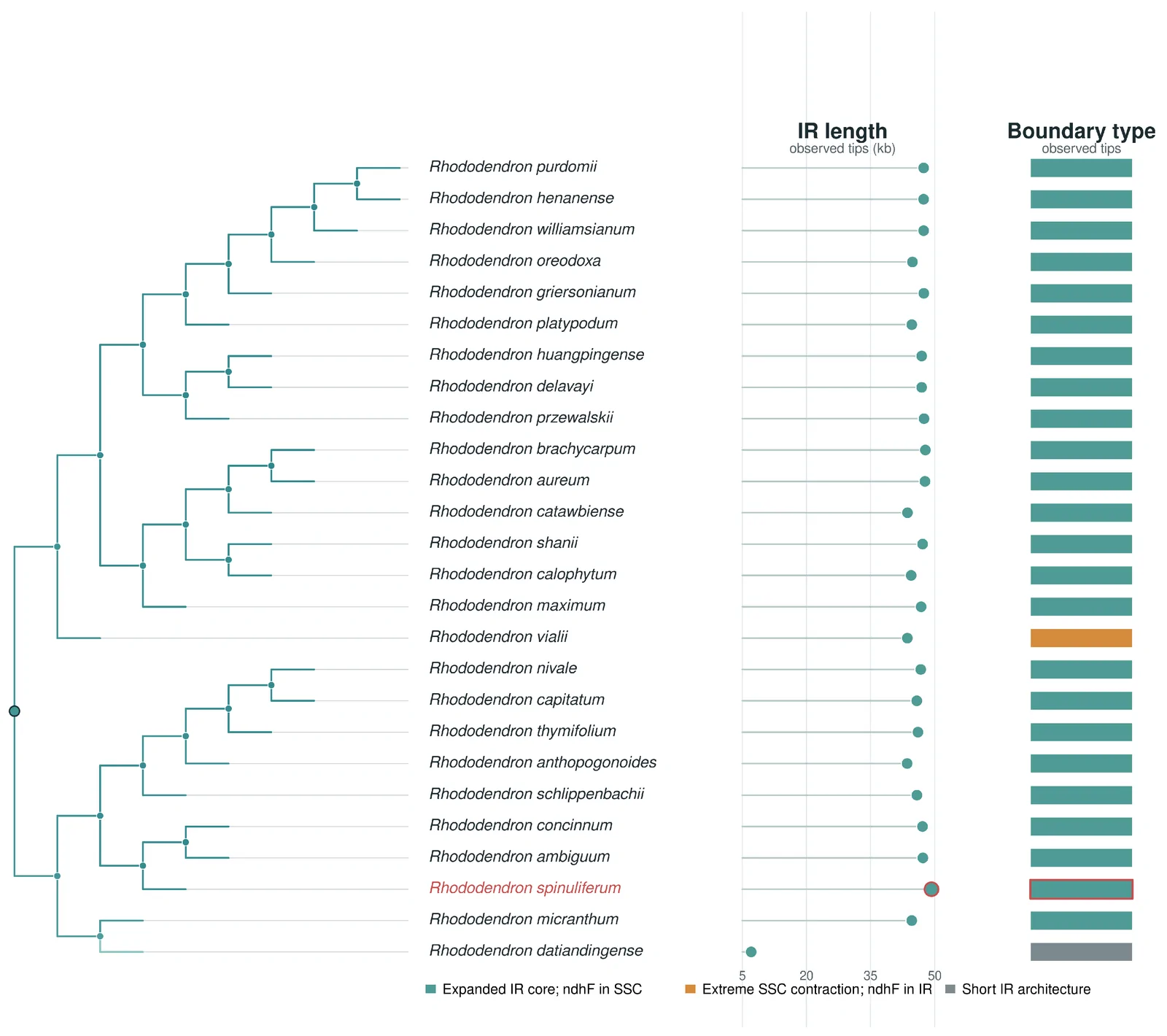
Phylogenetic mapping of IR length and validated boundary architecture in 26 structurally resolved *Rhododendron* plastomes. Branch and internal-node colours show the maximum-likelihood reconstruction of IR length; lollipops show observed tip values, and rectangles show the validated boundary type. *Rhododendron spinuliferum* is highlighted in red. The expanded-IR core with *ndhF* retained in the SSC was the most likely crown state (relative likelihood = 0.984). Continuous ancestral estimates are conditional on the fixed tree and root placement.

Phylogenetic mapping of the 26 structurally resolved plastomes showed that 24 (92.3%) retained the expanded-IR core with *ndhF* in the SSC; the remaining plastomes comprised the extreme SSC-contraction architecture of *R. vialii* and the short-IR architecture of *R. datiandingense*. Maximum-likelihood reconstruction identified the expanded-IR core with *ndhF* retained in the SSC as the most likely crown boundary state (relative likelihood = 0.984). The inferred crown values were 42.09 kb for IR length (95% CI: 41.33–42.85 kb) and 2.04 kb for SSC length (95% CI: 1.84–2.25 kb). *R. spinuliferum* had the longest IR among the sampled plastomes (49.266 kb) while retaining a typical SSC (2.629 kb), distinguishing its lineage-specific IR enlargement from the extreme SSC contraction in *R. vialii* (Figure 8). These intervals are therefore interpreted as conditional estimates rather than uncertainty ranges across alternative topologies or root placements. In the ingroup-only root-sensitivity check, the crown IR and SSC point estimates were 44.82 and 2.52 kb, respectively, while the expanded-IR core with *ndhF* retained in the SSC remained the most likely boundary state (relative likelihood = 0.980).

### 3.8. Repeat Landscape After Removal of One IR Copy

**Figure 9:**
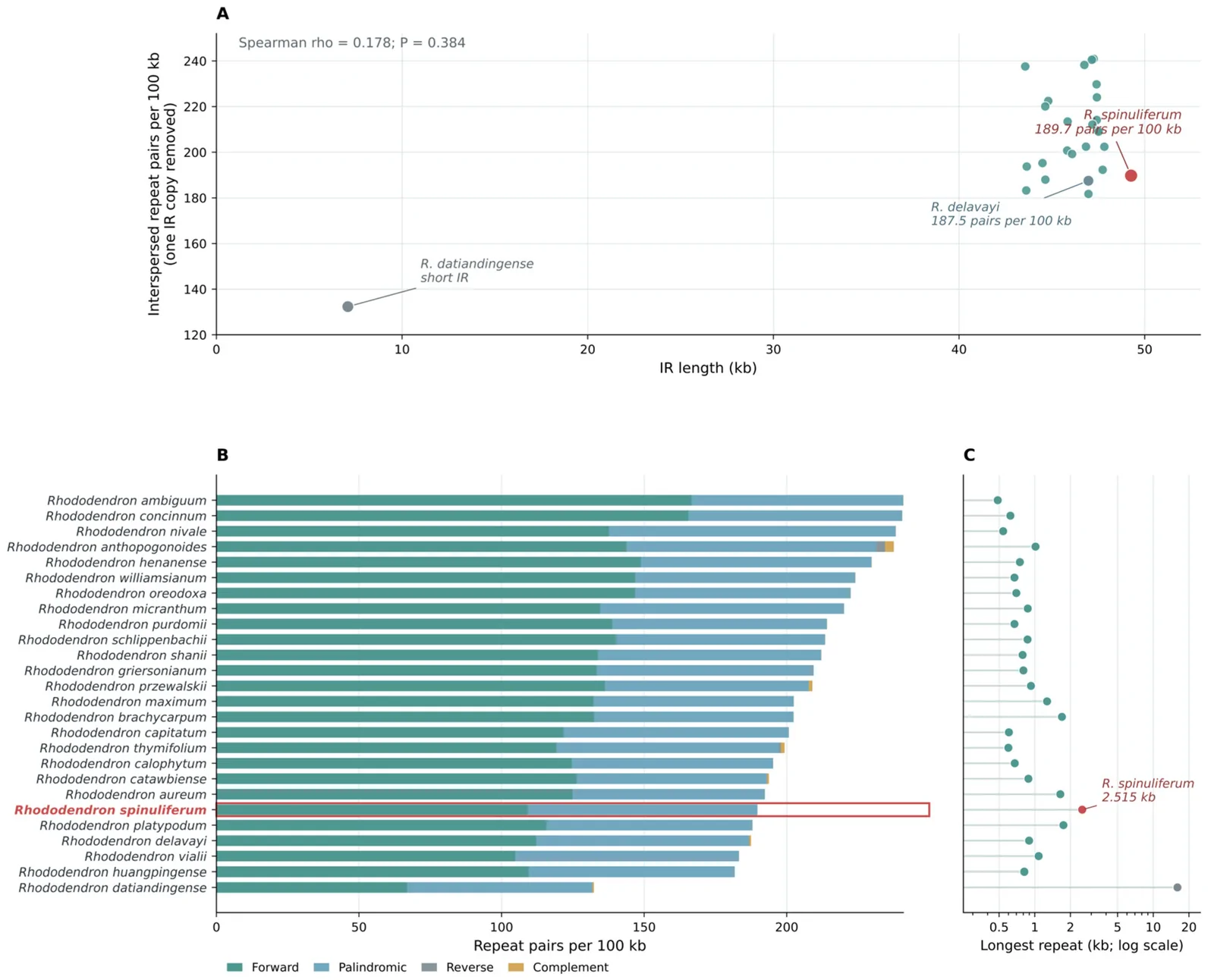
Interspersed-repeat landscape after removal of one validated IR copy from each of 26 *Rhododendron* plastomes. Vmatch was run with a minimum repeat length of 30 bp and up to three mismatches. No upper repeat-length limit was applied, and supermaximal repeats were retained. (**A**) Association between IR length and normalized repeat-pair density. (**B**) Repeat-pair density and orientation by taxon. (**C**) Longest retained repeat. *Rhododendron spinuliferum* is highlighted in red; *R. datiandingense* is the short-IR outlier. The red outline indicates the focal species, *Rhododendron spinuliferum*.

Across the 26 one-IR-removed plastomes, Vmatch identified 8517 nonredundant repeat pairs. IR length was not significantly associated with non-IR repeat-pair density (Spearman rho = 0.178, *p* = 0.384). This result indicates that no monotonic relationship was detected under the applied one-IR-removed Vmatch settings. *R. spinuliferum* contained 304 repeat pairs (189.7 pairs per 100 kb), similar to *R. delavayi* (291 pairs; 187.5 pairs per 100 kb). This 304-pair value, rather than the 217-pair short-repeat catalogue in Section 3.2, is the internally consistent value used for the 26-taxon comparison. Its longest retained repeat was 2515 bp, the largest among the expanded-IR taxa, and was largely noncoding (Figure 9).

### 3.9. Distributed Sequence Differences Underlie the Longer R. spinuliferum IR

**Figure 10:**
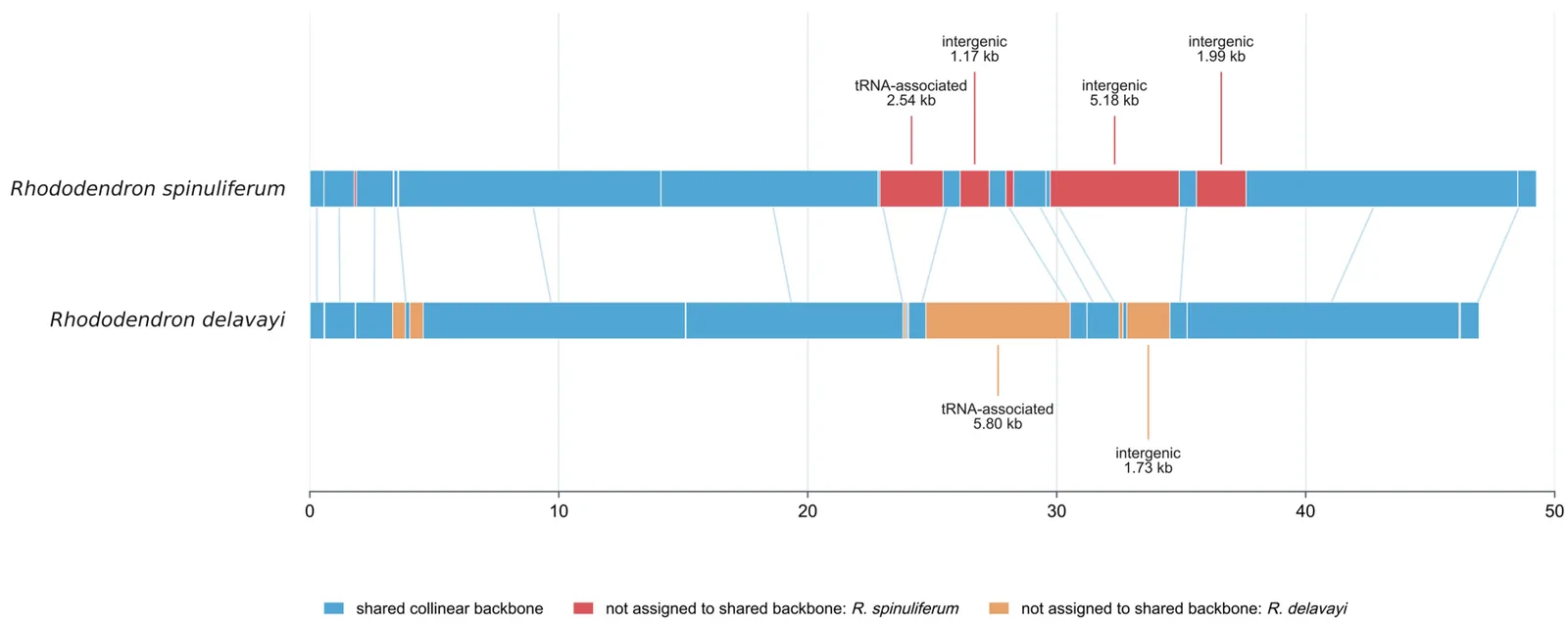
ProgressiveMauve alignment of the retained IR copies of *Rhododendron spinuliferum* and *R. delavayi*. Blue blocks denote the shared collinear backbone, and connecting lines link corresponding blocks. Red and orange blocks denote sequence not assigned to the shared backbone in *R. spinuliferum* and *R. delavayi*, respectively. Labels identify major non-collinear segments of at least 1 kb.

The 2298 bp IR-length excess in *R. spinuliferum* relative to the long-read *R. delavayi* plastome did not indicate a single contiguous insertion or capture of an additional complete functional gene. ProgressiveMauve identified a shared collinear backbone of 37,906 bp in *R. spinuliferum* and 37,949 bp in *R. delavayi*. Sequence not assigned to this shared backbone accounted for 11,360 and 9019 bp, respectively, producing a net 2341 bp non-collinear contribution that was partially offset by a 43 bp backbone difference. Most *R. spinuliferum* non-collinear blocks were intergenic, whereas one 2535 bp block was tRNA-associated. These results support distributed, predominantly intergenic sequence variation rather than a single extra-gene-capture event (Figure 10).

## 4. Discussion

The principal advance of this study is to convert an unusual plastome size into a read-supported structural inference. This distinction is important in repeat-rich plastomes, for which a circularized sequence can conceal an unresolved repeat graph or an incorrectly assigned junction. The long-read assembly of *R*. *delavayi* demonstrated both an expanded IR and evidence of plastid recombination [3]. Our graph curation and junction-spanning HiFi reads provide analogous structural evidence for *R. spinuliferum*. Because the Illumina reads came from a different individual, this evidence is interpreted as species-level support for the junction architecture rather than direct validation of the assembled HiFi individual. The additional SSC-orientation screen supported local IR-SSC-IR continuity, but the present read set could not quantify a reliable biological SSC-isomer ratio. Thus, the 49,266 bp IR is unlikely to be solely an assembly artefact. This evidence does not exclude intraspecific structural variation or heteroplasmy. Indeed, IR expansion can vary within and between populations in other plant lineages [32]. It does, however, establish a robust reference structure for comparative interpretation.

The sampled-taxon survey changes the interpretation of a long IR from an isolated peculiarity to a component of a predominant *Rhododendron* architecture. Most retained plastomes shared a validated 19-gene expanded-IR core while retaining *ndhF* in the SSC. The high likelihood assigned to this state at the crown node of the sampled tree is consistent with an early IR expansion followed by lineage-specific modification, rather than repeated origin of the same complete configuration. This interpretation places earlier reports of long IRs and short SSCs in a broader context [3,5,6,7]. Importantly, *R. spinuliferum* retained the common SSC arrangement despite having the longest validated conventional IR in the curated dataset. Its structure is therefore distinct from the near-complete SSC contraction in *R. vialii*, where *ndhF* is duplicated in the IR, and from the short-IR architecture of *R. datiandingense*. IR length and SSC length should consequently not be treated as a single interchangeable measure of plastome structural change. Because the continuous ancestral length estimates showed root-placement sensitivity, the evolutionary interpretation emphasizes the sampled boundary-state pattern rather than the exact reconstructed crown IR length.

Comparison with the long-read *R. delavayi* plastome further refines the explanation for the exceptional IR of *R. spinuliferum*. The approximately 2.3 kb difference was distributed across mostly intergenic and tRNA-associated sequence rather than concentrated in a single insertion or an additional complete protein-coding gene. In addition, repeat-pair density after removal of one IR copy was not correlated with IR length, and *R. spinuliferum* and *R. delavayi* had similar normalized repeat densities. These observations do not contradict the extensive repeats and recombination reported for *R. delavayi* [3]. Instead, they indicate that total repeat abundance alone is not a sufficient explanation for IR expansion in the sampled dataset. The identity, orientation, and genomic position of particular repeats may be more relevant than their aggregate number. The long noncoding repeat in *R. spinuliferum* may also explain why short reads were insufficient for primary resolution, but the present data do not establish it as the causal origin of IR expansion.

The potential biological consequences of IR expansion require equally careful interpretation. In some lineages, genes translocated into the IR show reduced substitution rates and elevated GC content, consistent with IR-mediated sequence homogenization or gene conversion [33]. Broader comparisons have also associated IR dynamics with rate variation [34]. However, IR expansion did not reduce substitution rates in *Pelargonium*, demonstrating that this relationship is lineage-dependent [35]. In *R. spinuliferum*, the additional IR sequence was predominantly noncoding and did not reflect capture of a new complete functional gene. The Ka/Ks analysis also did not provide robust evidence for positive selection. Therefore, the data do not justify assigning an adaptive advantage, altered gene dosage, or a high-altitude response to the long IR. Likewise, high Pi values near the very short SSC and boundary-associated loci should not automatically be interpreted as conventional hypervariable markers, because window estimates in a structurally constrained region can be disproportionately influenced by a small number of boundary-proximal differences. This interpretation is reinforced by the fact that only 11 windows were fully contained within the 2629 bp SSC, while 6 additional windows adjacent to the SSC crossed an IR/SSC boundary and were analyzed as junction windows.

The structural results also have implications for plastome-based systematics and for the curation of public resources. The coding-sequence tree placed *R. spinuliferum* with *R. schlippenbachii*, whereas the shared expanded-IR core spans much more deeply across the sampled *Rhododendron* phylogeny. Boundary architecture can therefore complement sequence-based phylogenetic evidence as a diagnostic structural character, but it should not be used as a substitute for a species tree. The densely sampled plastome phylogeny of *Rhododendron* also recovered discordance between plastid and nuclear histories [4], a reminder that plastome traits represent an organellar evolutionary history. The exclusion of seven publicly available records with unresolved IR/SSC structures was thus a substantive analytical decision rather than a technical convenience. Including them without validation could have converted annotation or assembly uncertainty into an apparent evolutionary pattern.

Several limitations define the scope of the present conclusions. The 26 validated plastomes represent only a fraction of the diversity of *Rhododendron*, and taxon sampling remains uneven among sections. The comparison also cannot determine whether the long-IR configuration segregates within populations, nor can sequence data alone measure transcriptional or physiological consequences. Long-read population sampling, complete nuclear phylogenomic context, and targeted expression analyses will be needed to address these questions. Nevertheless, this study moves beyond reporting another large plastome. It provides a validated framework in which the exceptionally long IR of *R. spinuliferum* can be interpreted as a lineage-specific extension of a widespread expanded-IR background, dominated by distributed noncoding variation rather than a simple increase in repeat abundance or complete gene capture.

## 5. Conclusions

By integrating long-read graph resolution, junction-spanning read support, and independent short-read structural support, we established a robust plastome reference for *R*. *spinuliferum*. Its 49,266 bp IR is the longest validated conventional IR in the curated, structurally resolved dataset, but it retains the common SSC configuration rather than the extreme SSC contraction observed in *R. vialii*. The 26-taxon survey supports a widespread expanded-IR core as the likely crown state within the sampled *Rhododendron* lineages. Comparison with *R. delavayi* further indicates that the additional IR sequence is distributed and predominantly noncoding, whereas repeat abundance after removal of one IR copy does not explain IR length. Thus, the principal contribution is a validated evolutionary framework: long IRs in *Rhododendron* should be interpreted through boundary architecture and sequence composition, not genome size alone. Broader long-read sampling is needed to test the population-level stability and functional consequences of this configuration.

## Figures and Tables

**Table 1:** Gene composition in the plastome of *Rhododendron spinuliferum*.

Category	Subcategory	Genes	No. of Genes
Photosynthesis	Photosystem I	*psaA*, *psaB*, *psaC* †, *psaI* †, *psaJ*	7
	Photosystem II	*psbA*, *psbB*, *psbC*, *psbD*, *psbE*, *psbF*, *psbH*, *psbI*, *psbJ*, *psbK*, *psbL*, *psbM*, *psbN*, *psbT*, *psbZ*	15
	NADH dehydrogenase	*ndhA* * †, *ndhB* *, *ndhC*, *ndhD* †, *ndhE* †, *ndhF*, *ndhG* †, *ndhH* †, *ndhI* †, *ndhJ*, *ndhK*	17
	Cytochrome b6/f complex	*petA* †, *petB* *, *petD* *, *petG*, *petL*, *petN*	7
	ATP synthase	*atpA*, *atpB*, *atpE*, *atpF* *, *atpH*, *atpI*	6
	Rubisco large subunit	*rbcL*	1
Genetic apparatus	Ribosomal proteins (LSU)	*rpl2*, *rpl14*, *rpl16* *, *rpl20*, *rpl22*, *rpl23*, *rpl32* †, *rpl33*, *rpl36*	10
	Ribosomal proteins (SSU)	*rps2*, *rps3* †, *rps4*, *rps7*, *rps8*, *rps11*, *rps12* **, *rps14*, *rps15* †, rps1 6* †, *rps18*, *rps19* †	16
	RNA polymerase	*rpoA*, *rpoB* *, *rpoC1*, *rpoC2*	4
	rRNAs	*rrn4.5* †, *rrn5* †, *rrn16* †, *rrn23* †	8
	tRNAs	*trnA-UGC* * †, *trnArg-ACG* †, *trnArg-TCT*, *trnAsn-GTT* †, *trnAsp-GTC*, *trnCys-GCA*, *trnGln-TTG*, *trnGlu-TTC*, *trnGly-GCC*, *trnHis-GTG*, *trnIle-GAT* †, *trnLeu-CAA*, *trnLeu-TAG* †, *trnMet-CAT* †, *trnPhe-AAA* †, *trnPhe-GAA*, *trnPro-TGG*, *trnSer-GCT*, *trnSer-GGA*, *trnSer-TGA*, *trnThr-GGT*, *trnThr-TGT*, *trnTrp-CCA*, *trnTyr-GTA*, *trnVal-GAC* †	41
Other/housekeeping	Misc.	*ccsA* †, *cemA* †, *infA*, *matK*	6
	Conserved hypothetical chloroplast ORFs	*ycf1* †, *ycf2*, *ycf3* **, *ycf4* †	8
Total			146

* Gene with one intron; ** gene with two introns. Gene copies in IRa and IRb are counted separately. † Gene present in two or more copies.

## Data Availability

The complete chloroplast genome sequence of *R. spinuliferum* generated in this study is available in GenBank at the National Center for Biotechnology Information (NCBI; https://www.ncbi.nlm.nih.gov) under accession number PZ603433. This GenBank sequence was assembled from the public PacBio HiFi run CRR1094297 and was not derived from the same source individual as voucher LSU_20260312. The Illumina paired-end reads generated from the Zixi Mountain voucher individual for independent structural support are available in the NCBI Sequence Read Archive under accession SRR38226386, BioProject PRJNA1303833, and BioSample SAMN57403931. The publicly available PacBio HiFi reads used for the primary de novo assembly were retrieved from the Genome Sequence Archive at the National Genomics Data Center (NGDC; https://ngdc.cncb.ac.cn) under accession CRR1094297.

## Supplementary Materials

The following supporting information can be downloaded at: https://www.mdpi.com/article/10.3390/genes17091007/s1, Figure S1: Assembly graph interpretation and structural support for the *Rhododendron spinuliferum* plastome. (A) Bandage rendering of the Flye assembly graph. (B) The manually resolved circular path yielded a 209,482-bp plastome with two 49,266-bp IRs, a 108,321-bp LSC, and a 2,629-bp SSC. (C) PacBio HiFi reads spanning at least 1 kb on both sides of each synthetic junction. (D) PE400 reads from a different individual spanning at least 50 bp on both sides of each junction. The PE400 reads were used for species-level structural support only and were not used to polish the HiFi-derived sequence. Figure S2: Whole-plastome read coverage supporting the manually resolved *Rhododendron spinuliferum* plastome. Per-base read depth was calculated with samtools and summarized in non-overlapping 500-bp bins. Shaded bands indicate IRa, LSC, IRb, and SSC, and dashed lines mark the junctions. (A) PacBio HiFi reads recruited for primary assembly. (B) Independently generated Illumina PE400 reads from a different *R. spinuliferum* individual. PE400 mapping covered all 209,482 positions, with no zero-depth bases (median depth, 578x; mean depth, 572.52x), and was used for independent species-level structural support only. The PE400 depth values were generated by mapping reads to the final manually curated 209,482-bp circular plastome assembly deposited as PZ603433, rather than to an intermediate assembly. Figure S3: Relative synonymous codon usage (RSCU) in the *Rhododendron spinuliferum* plastome. Codons are grouped by encoded amino acid and colored according to their position within each synonymous-codon family. Table S1: Taxa and accessions used in comparative and phylogenetic analyses. Table S2: Public *Rhododendron* plastome records excluded from the within-genus IR/SSC structural survey.
