# Supplementary material for: Long-Read Assembly and a Within-Genus Structural Survey Reveal an Exceptionally Long Inverted Repeat in the Rhododendron spinuliferum Plastome
Source: Genes (Basel). 2026 Aug 26;17(9):1007. doi: 10.3390/genes17091007 (PMC13606625; doi:10.3390/genes17091007)
Supplement: Supplementary file 1 [file genes-17-01007-s001.zip › genes-4488805-supplementary.pdf]

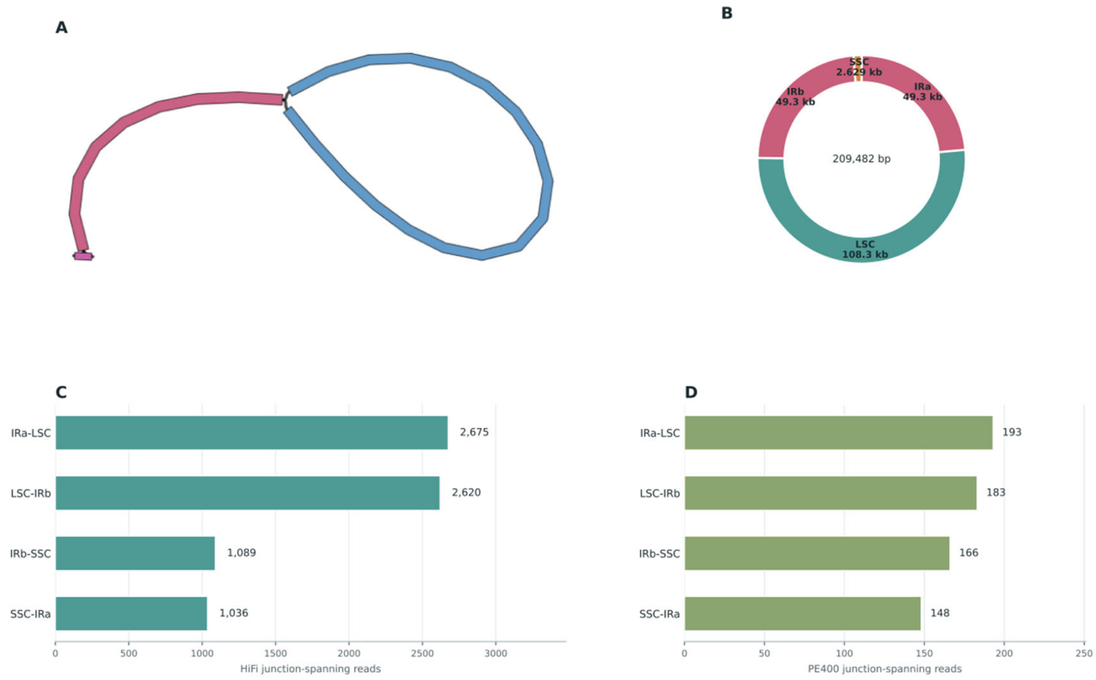

Figure S1. Assembly graph interpretation and structural support for the *Rhododendron spinuliferum* plastome. (A) Bandage rendering of the Flye assembly graph. (B) The manually resolved circular path yielded a 209,482-bp plastome with two 49,266-bp IRs, a 108,321-bp LSC, and a 2,629-bp SSC. (C) PacBio HiFi reads spanning at least 1 kb on both sides of each synthetic junction. (D) PE400 reads from a different individual spanning at least 50 bp on both sides of each junction. The PE400 reads were used for species-level structural support only and were not used to polish the HiFi-derived sequence.

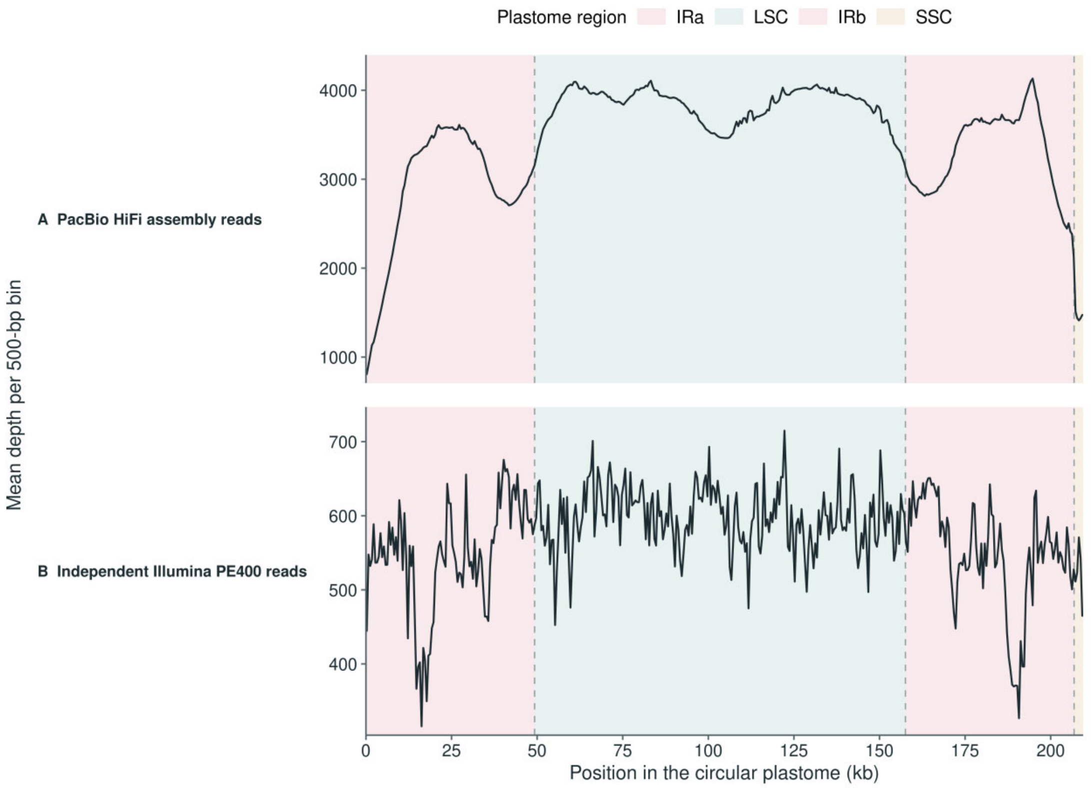

Figure S2. Whole-plastome read coverage supporting the manually resolved *Rhododendron spinuliferum* plastome. Per-base read depth was calculated with samtools and summarized in non-overlapping 500-bp bins. Shaded bands indicate IRa, LSC, IRb, and SSC, and dashed lines mark the junctions. (A) PacBio HiFi reads recruited for primary assembly. (B) Independently generated Illumina PE400 reads from a different *R. spinuliferum* individual. PE400 mapping covered all 209,482 positions, with no zero-depth bases (median depth, 578x; mean depth, 572.52x), and was used for independent species-level structural support only. The PE400 depth values were generated by mapping reads to the final manually curated 209,482-bp circular plastome assembly deposited as PZ603433, rather than to an intermediate assembly.

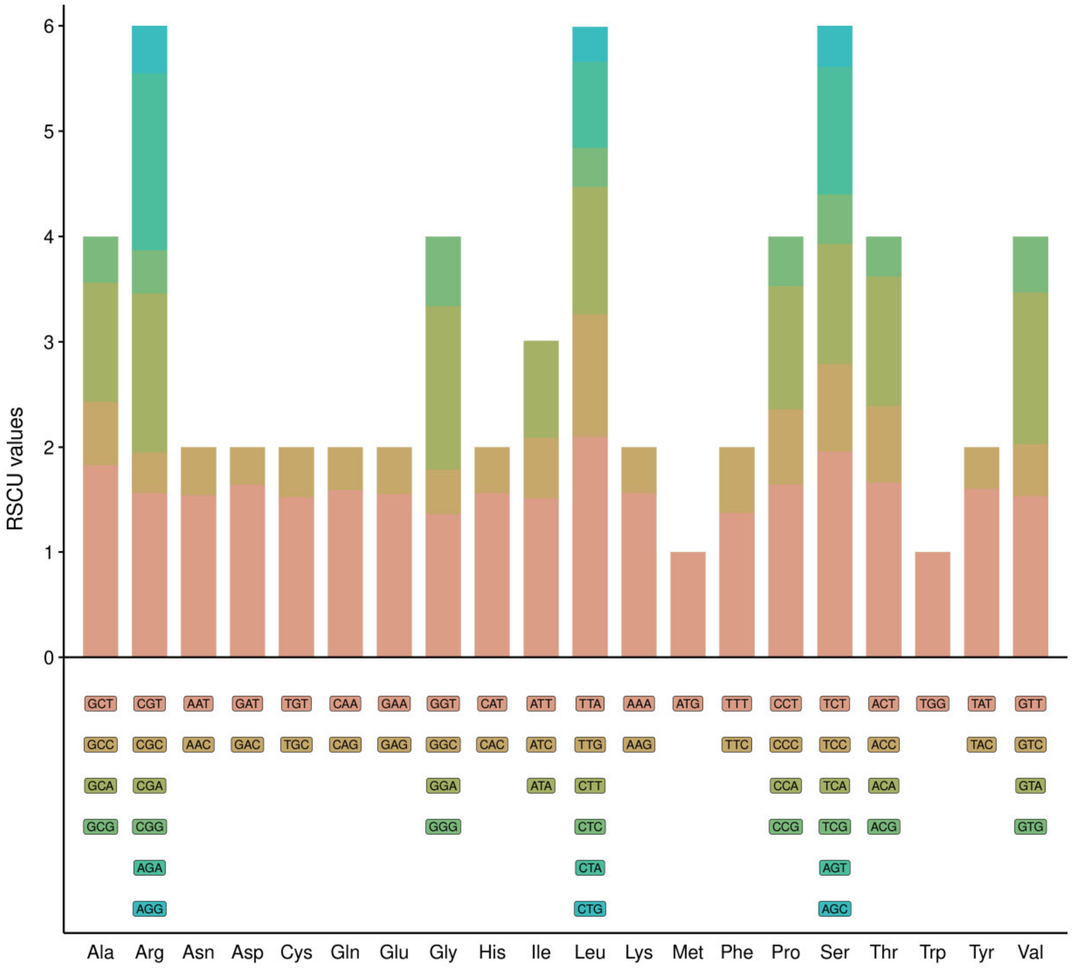

Figure S3. Relative synonymous codon usage (RSCU) in the *Rhododendron spinuliferum* plastome. Codons are grouped by encoded amino acid and colored according to their position within each synonymous-codon family.

Table S1. Taxa and accessions used in comparative and phylogenetic analyses.

Scientific name	Accession	Role
Rhododendron spinuliferum	PZ603433	ingroup
Rhododendron ambiguum	OR455462	ingroup
Rhododendron anthopogonoides	OL871191	ingroup
Rhododendron aureum	OM574972	ingroup
Rhododendron brachycarpum	OM574995	ingroup
Rhododendron calophytum	OM373082	ingroup
Rhododendron capitatum	OL804295	ingroup
Rhododendron catawbiense	OM575017	ingroup
Rhododendron concinnum	MT239366	ingroup
Rhododendron datiandingense	MW381788	ingroup
Rhododendron delavayi	MN413198	ingroup
Rhododendron delavayi	MN711645	Figure 10, long-IR comparison
Rhododendron farrerae	OQ980386	ingroup
Rhododendron griersonianum	MT533181	ingroup
Rhododendron henanense	MT239363	ingroup
Rhododendron huadingense	OM177184	ingroup
Rhododendron huangpingense	PZ519590	ingroup
Rhododendron kawakamii	MW762686	ingroup
Rhododendron latoucheae	OL782150	ingroup
Rhododendron maximum	OM575018	ingroup
Rhododendron micranthum	MZ457925	ingroup
Rhododendron molle	MZ073672	ingroup
Rhododendron nivale	PQ536991	ingroup
Rhododendron oreodoxa	OL639014	ingroup
Rhododendron ovatum	OR129260	ingroup
Rhododendron platypodum	MT985162	ingroup
Rhododendron przewalskii	OL871190	ingroup
Rhododendron purdomii	OP279445	ingroup
Rhododendron schlippenbachii	OM575019	ingroup
Rhododendron shanii	MW374796	ingroup
Rhododendron simsii	MW030509	ingroup
Rhododendron thymifolium	OL804294	ingroup
Rhododendron vialii	CP127078	ingroup
Rhododendron williamsianum	PP690644	ingroup
Gaultheria nummularioides	OL944386	outgroup
Vaccinium bracteatum	PQ284534	outgroup

Table S2. Public *Rhododendron* plastome records excluded from the within-genus IR/SSC structural survey.

Scientific name	Accession screened	Genome length (bp)	Specific reason for exclusion
Rhododendron farrerae	NC_082049	149,453	No confidently resolved pair of inverted repeats was detected by whole-plastome BLAST+ self-alignment.
Rhododendron huadingense	NC_063113	198,952	No confidently resolved pair of inverted repeats was detected by whole-plastome BLAST+ self-alignment.
Rhododendron kawakamii	NC_058233	230,777	GenBank IR annotation was not verified as a single conventional inverted-repeat pair by whole-plastome BLAST+ self-alignment.
Rhododendron latoucheae	NC_063094	156,355	No confidently resolved pair of inverted repeats was detected by whole-plastome BLAST+ self-alignment.
Rhododendron molle	MZ073672	197,877	No confidently resolved pair of inverted repeats was detected by whole-plastome BLAST+ self-alignment.
Rhododendron ovatum	OR129260	195,987	No confidently resolved pair of inverted repeats was detected by whole-plastome BLAST+ self-alignment.
Rhododendron simsii	NC_053764	152,214	No confidently resolved pair of inverted repeats was detected by whole-plastome BLAST+ self-alignment.

Note: accession screened refers to the representative public record selected for the species-level IR/SSC structural screening.
